# Assessing the left atrial function via echocardiography to detect cirrhotic cardiomyopathy in patients with liver cirrhosis - a scoping review

**DOI:** 10.1186/s12947-026-00383-5

**Published:** 2026-10-01

**Authors:** M Swathi Poojary, Shiran Shetty, Jyothi Samanth, Mahidhar Jeedigunta, Vennila Jaganathan, Shubha Srinivas, Monika P Jawanjal, Sridevi Prabhu, Vidya Nayak, Krishnananda Nayak

**Affiliations:** 1 Department of Cardiovascular Technology, Manipal College of Health Professions, Manipal Academy of Higher Education, Manipal, Karnataka 576104 India; 2 Department of Gastroenterology, Kasturba Medical College, Manipal, Manipal Academy of Higher Education, Manipal, Karnataka 576104 India; 3 Department of Cardiology, Kasturba Medical College, Manipal, Manipal Academy of Higher Education, Manipal, Karnataka 576104 India; 4 Faculty of Statistics, Manipal College of Health Professions, Manipal Academy of Higher Education, Manipal, Karnataka 576104 India

## Abstract

**Background:**

Cirrhotic cardiomyopathy (CCM) is a condition observed in patients with chronic liver disease, characterized by dysfunction in both cardiac diastolic and/or systolic function. This condition is often underdiagnosed due to a lack of awareness and the possibility of misdiagnosis. Consequently, there is limited data regarding subclinical CCM in individuals with end-stage liver disease. Furthermore, there is currently no established cardiac biomarker to assess cardiovascular risk and outcomes in patients with liver cirrhosis. To address this issue, we conducted a scoping review to map the existing evidence and identify research gaps in echocardiographic assessment of left atrial (LA) function for detecting CCM in patients with liver cirrhosis.

**Methods:**

We systematically searched the Scopus, PubMed, and Web of Science databases using Population, Concept, and Context framework. The population is adult patients with liver cirrhosis. The concept is to assess LA function via echocardiography. Context is to detect CCM.

**Results:**

We identified 364 articles, of which 24 were eligible for full-text review. Ultimately, 20 articles were included in the final analysis. Our dataset revealed various cardiovascular manifestations involving LA. Key findings related to CCM included LA enlargement and increased LA diameter and volume, which correlated with the Child-Turcotte-Pugh and Model for End-Stage Liver Disease scores. Additionally, we observed a reduction in LA strain. LA diameter, volume, and LA strain were associated with outcome prediction.

**Conclusion:**

Evaluating LA function with advanced echocardiographic techniques offers a promising approach for early detection and prognostic assessment of CCM. To translate these findings into standardized clinical practice, bridging existing evidence gaps with well-designed studies is crucial.

**Graphical abstract:**

**Figure:**
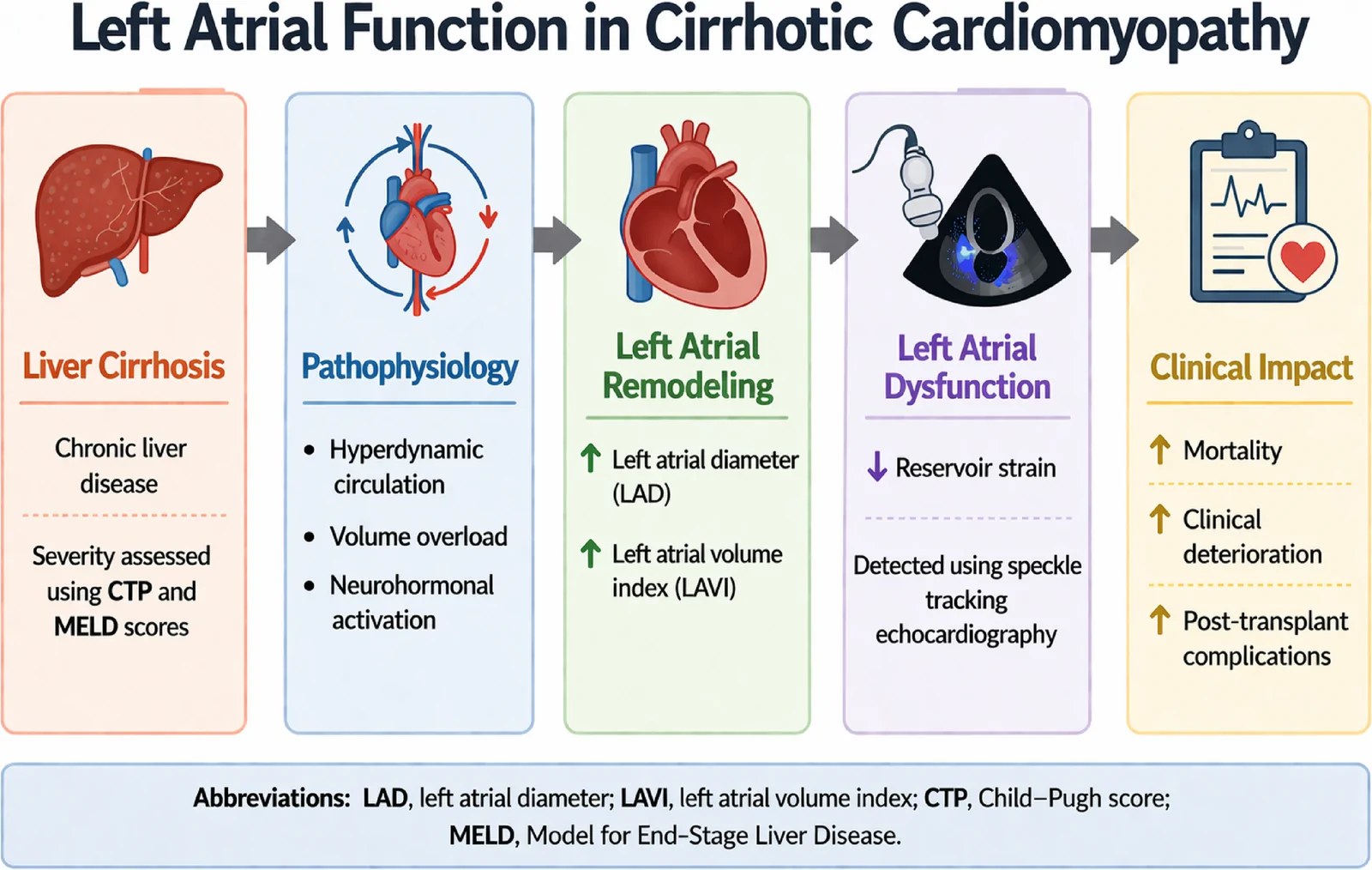

**Supplementary Information:**

The online version contains supplementary material available at https://doi.org/10.1186/s12947-026-00383-5.

## What is already known on this topic

CCM is a hyperdynamic condition that causes subclinical cardiac dysfunction in patients with liver cirrhosis.

Major guidelines for CCM lack a clear definition and consistent diagnostic criteria.

## What this study adds

This scoping review provides the most up-to-date and comprehensive literature on assessing LA function using echocardiography to detect CCM.

## How this study might affect research, practice, or policy

There is a need for further prospective research to validate advanced echocardiographic techniques and clarify the correlation between liver and cardiac disease severity. Also, there is a need for an update in the guidelines for the management of cirrhotic patients, particularly those awaiting liver transplantation or undergoing invasive procedures.

## Background

**Fig. 1:**
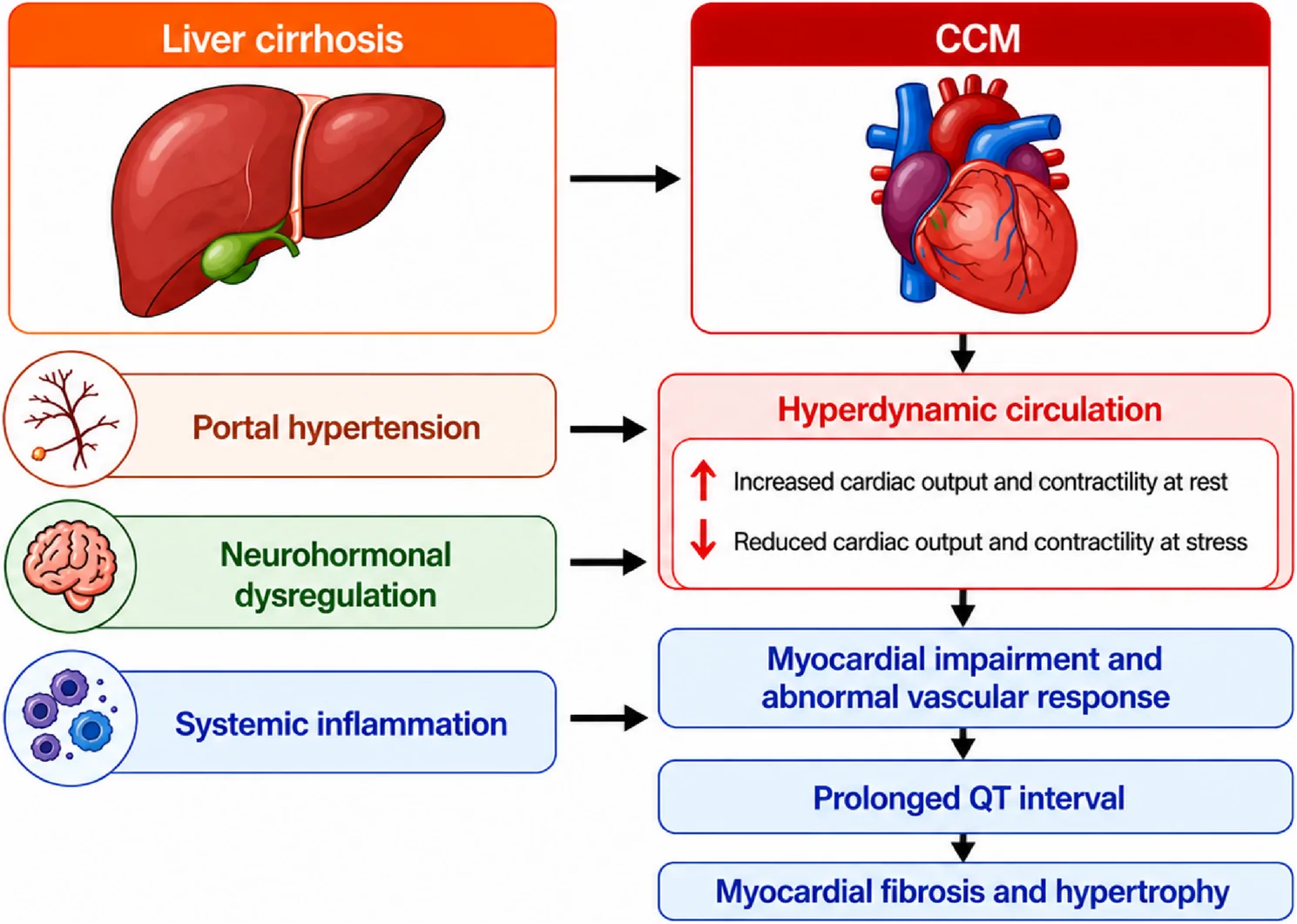
Pathophysiology of CCM. *Abbreviations*: *CCM* Cirrhotic Cardiomyopathy

**Fig. 2:**
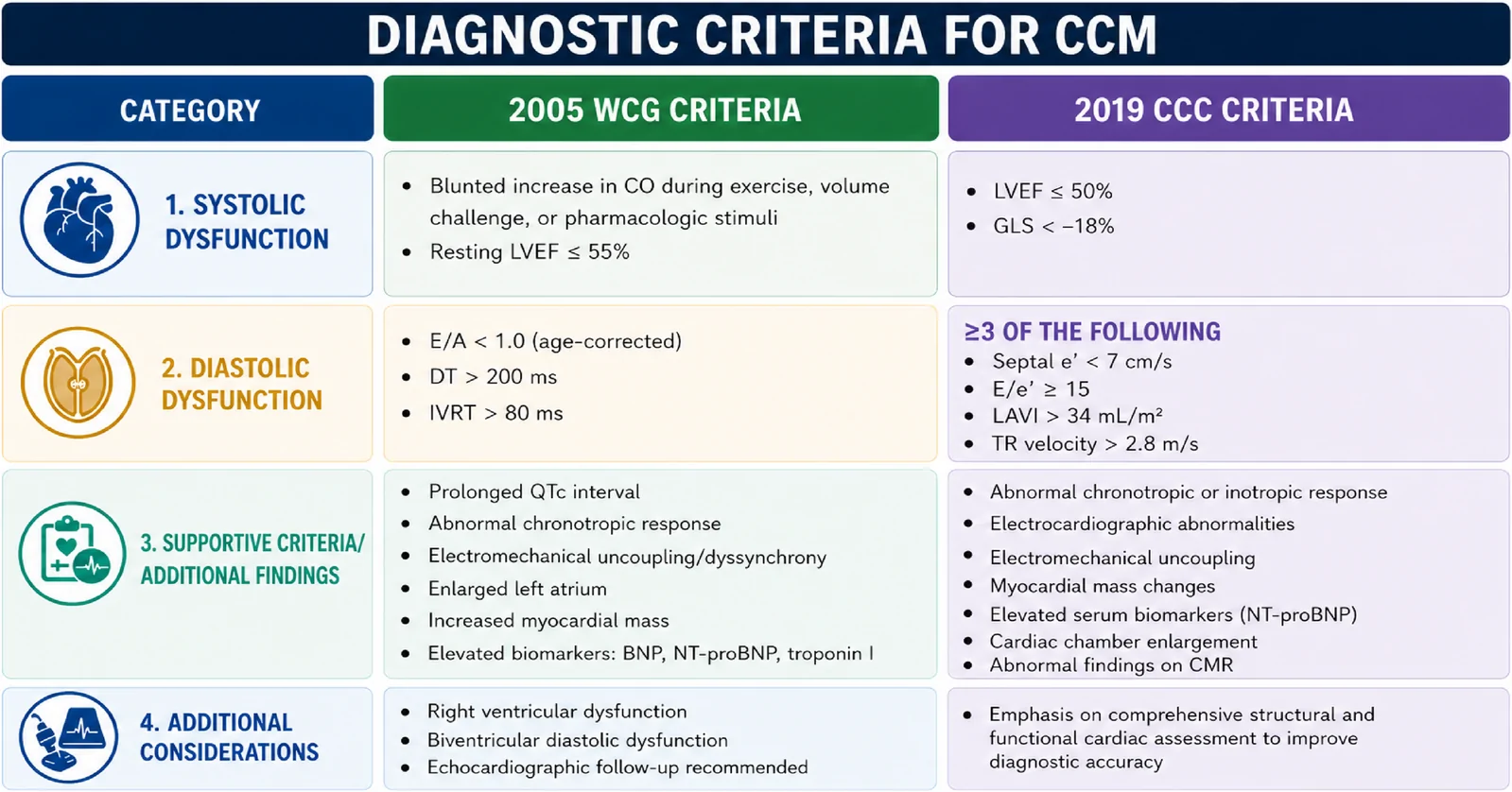
Diagnostic Criteria for CCM (2005 WCG & 2019 CCC Updates) [4–10]. *Abbreviations*: *CCM* Cirrhotic Cardiomyopathy, *WCG* World Congress of Gastroenterology, *CCC* Cirrhotic Cardiomyopathy Consortium, *CO* Cardiac Output, *LVEF* Left Ventricular Ejection Fraction, *GLS* Global Longitudinal Strain, *E/A* Mitral Early-to-Late Filling Ratio, *DT* Deceleration Time, *IVRT* Isovolumetric Relaxation Time, *e*′ Early Diastolic Mitral Annular Velocity, *E/e*′ Ratio of Early Mitral Inflow Velocity to Mitral Annular Velocity, *TR* Tricuspid Regurgitation, *LAVI* Left Atrial Volume Index, *QTc* Corrected QT Interval, *BNP* B-type Natriuretic Peptide, *NT-proBNP* N-terminal pro–B-type Natriuretic Peptide, *CMR* Cardiac Magnetic Resonance

Cirrhotic cardiomyopathy (CCM) is a chronic cardiac dysfunction in patients with liver cirrhosis, regardless of the aetiology of liver cirrhosis, with the mechanism unrelated to preexisting structural heart diseases [1]. CCM features include impaired left ventricular (LV) systolic function in response to stress, diastolic dysfunction (DD), and electrophysiological abnormalities [2] (Fig. 1). The World Congress of Gastroenterology (WCG) initially established diagnostic criteria for CCM in 2005 [3]. The Cirrhotic Cardiomyopathy Consortium (CCC) introduced updated criteria in 2019, derived from advanced echocardiographic techniques such as tissue Doppler imaging and myocardial strain [4–10] (Fig. 2).

It is essential to detect early cardiac changes in cirrhotic patients, which is crucial for managing CCM and improving outcomes, especially in patients who are undergoing liver transplantation (LT). Using advanced echocardiographic techniques, such as Speckle Tracking Echocardiography (STE) and Three-Dimensional Echocardiography (3DE), provides a detailed understanding of cardiac involvement in liver cirrhosis [11]. Left Atrial (LA) function, particularly LA size, is frequently altered in liver disease, with enlargement correlating with disease severity, blood volume changes, and systemic hemodynamic alterations. These findings highlight the interconnected nature of cardiac and hepatic dysfunction and suggest that LA measurements may provide valuable insights into the cardiovascular impact of liver disease [12–15].

This scoping review aims to provide a comprehensive overview of echocardiographic techniques and their effectiveness in assessing LA function in CCM. It systematically maps the existing literature, highlighting key echocardiographic parameters, their association with liver disease severity, and their prognostic significance. Additionally, it identifies gaps in current evidence and areas requiring further investigation to enhance clinical understanding and future research directions.

## Methods

### Research question

How does echocardiographic assessment of LA function contribute to the early detection and characterization of CCM in patients with liver cirrhosis?

### Protocol and registration

Our scoping review strategy was based on the hypothesis that LA structural changes are prevalent in patients with liver cirrhosis and correlate positively with the severity of liver dysfunction, as measured by the Model for End-Stage Liver Disease (MELD) or Child-Pugh-Turcotte (CTP) scores. The review aligns with the Joanna Briggs Institute (JBI) guidance and is reported according to the Preferred Reporting Items for Systematic Reviews and Meta-Analyses Extension for Scoping Review (PRISMA-ScR) standard [16]. The study protocol and search strategy were registered on the Open Science Framework (DOI: https://doi.org/10.17605/OSF.IO/YB65M). A checklist is included in the online supplemental files.

### Search strategy, sources, and eligibility criteria

Using an initial focused search, we identified key papers on the topic to devise a comprehensive search strategy in conjunction with an experienced librarian. We subsequently searched PubMed, Scopus, and Web of Science using the Population, Concept, and Context (PCC) framework outlined by the JBI for scoping reviews [17]. The search strategy, including text words and subject headings, was adapted for each database. The reference list of all included sources of evidence was screened for additional eligible articles. Population: The scoping review focused on adult patients with liver cirrhosis. Concept: Assessment of LA function via echocardiography. Context: detecting CCM (Table 1). Studies were included if they reported the predefined outcomes of interest, namely left atrial structural and functional parameters.

**Table 1:**
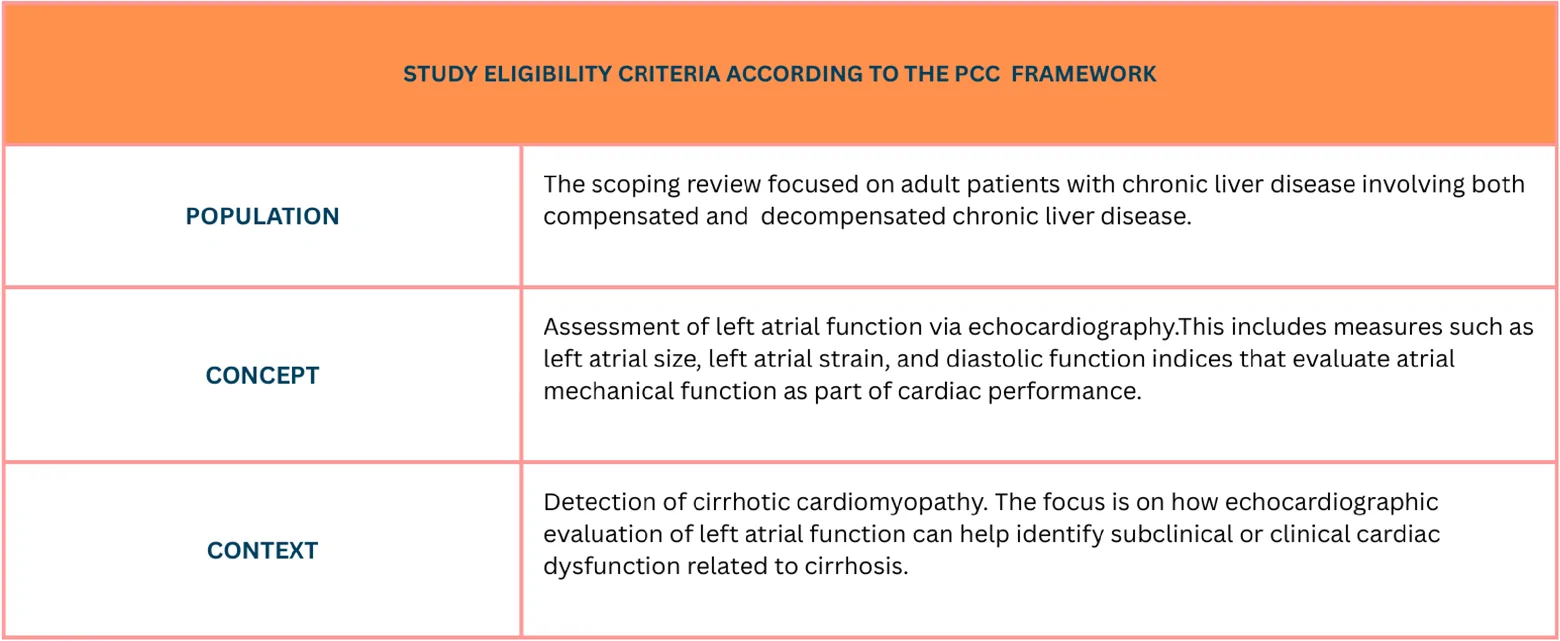
Study eligibility criteria according to the PCC framework

*Abbreviations*: *PCC* Population Concept Context

### Sources and selection of evidence

**Fig. 3:**
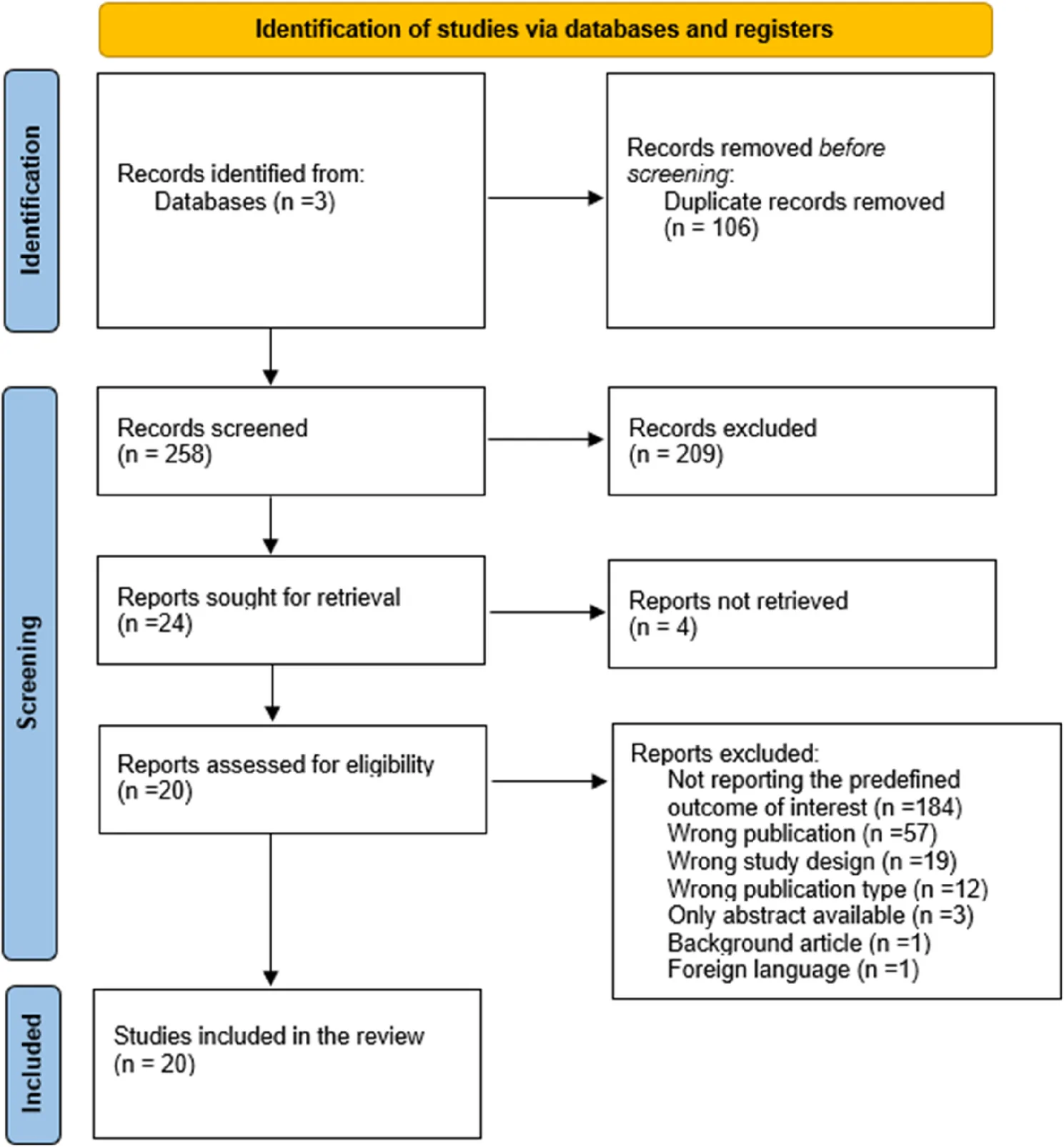
Flowchart of included studies

Prospective and retrospective observational, cohort, cross-sectional, or case-control studies were included. Reviews, conference proceedings, conference abstracts, or book chapters were not reviewed to avoid duplication that may have occurred in published studies. Following the search, all identified citations were collated and uploaded into the Rayyan platform, and duplicates were removed. Title/abstract and full-text screening were performed at two levels. Two reviewers were included. Reviewers were blinded at the initial screening stage. The process of resolving disagreements was done by consensus agreement. The search results and the study inclusion process were reported using the PRISMA-ScR flow diagram (Fig. 3).

### Data extraction, data analysis & presentation

Data was extracted by two independent reviewers using the data extraction tool. The extracted data include specific details on the first author, year, country, sample numbers, methodology, CTP score, MELD score, aetiology of cirrhosis, LA function parameters assessed, key findings related to LA function and CCM, and outcome measures. The predefined outcomes of interest were LA structural and functional parameters, including LA diameter (LAD), LA volume (LAV), LA volume index (LAVI), LA strain, and other echocardiographic indices reflecting LA function. The form was pilot tested. Any discrepancies during the data extraction were resolved by discussion or with an additional reviewer. The authors identified gaps in the literature to formulate questions for further research. Findings were synthesized qualitatively, using descriptive tables to address the review objectives. Formal assessment of study quality, risk of bias, or publication bias was not conducted.

## Results

### Search results

The selection process is outlined in Fig. 3. In total, 364 articles were identified. From these articles, 258 were eligible for screening after duplicates were removed, and 20 were subsequently eligible for full-text review. A total of 184 records were excluded due to not reporting the predefined outcome of interest, 57 due to the wrong population, 19 due to the wrong study design, 12 due to the wrong publication type, 3 due to only an abstract available, 1 due to a background article, and 1 due to a foreign language.

### Study characteristics and methodological approaches

A summary of the review article is represented in the Table 2. A total of 20 studies were included in the scoping review, consisting of 15 prospective studies (case–control [7], cross-sectional [5], cohort [3], follow-up [1], observational [3]) and five retrospective studies (cohort [2], cross-sectional [1]). We have identified articles from 2014 to 2025. CCM research spans Asia (China, Korea, India, Pakistan), Europe (Italy, Greece, Turkey, Germany, Romania, Denmark, Serbia, Belgium), North Africa (Tunisia), and beyond. This global distribution enriches understanding of CCM’s clinical spectrum and regional aetiological patterns and advances diverse approaches to cardiac prognostic stratification in cirrhosis.

**Table 2:** Summary of the articles included in the scoping review

Sl. No	First author and year	Study period	Sample size(n)	Methodology	CTP Score MELD score(Mean/median/%)	Aetiology (n/%)	LA parameters assessed	Key findings related to LA function and CCM	Cardiac prognostic outcome
1	Voet et al.,2025,Belgium [18]	January 2005-March 2017	233-cirrhotic patients	Retrospective cohort	CTP: A (36), B (96), C (86); MELD: median 17 (IQR 11)	Alcohol-related (116),autoimmune/ biliary disease (37),HBV (23),HCV (33),MASLD (22)	E, A, e′(septal/lateral), a′ (septal/lateral), E/A, E/e′	Lower prevalence of DD with revised criteria; reduced lateral e′ associated with mortality	LA function and reduced e′ are associated with post-LT mortality
2	Lupu et al.,2024,Romania [19]	November 2021- March 2024	102-cirrhotic patients	Prospective observational	CTP A/B (n = 65), CTP C (n = 37)	Alcohol (77),viral (18),others (7)	LAVI, E/e′	Greater DD burden in advanced cirrhosis is correlated with portal hypertension severity	Not reported
3	Wang et al.,2024,China [20]	22 June 2020-30 June 2022	104-cirrhotic patients	Retrospective cohort	CTP: A/B (35.6%), C (64.4%); MELD: mean 11.94 ± 5.46	HBV (30),HCV (6),alcohol (47)	LAD, E, A, E/A	E/A ≤ 0.8 is independently associated with mortality	E/A ≤ 0.8 predicts higher mortality
4	Mansoor et al.,2024,Pakistan [21]	August 2021-June 2023	68-cirrhotic patients	Prospective cross-sectional	CTP: A (32.4%), B (57.4%), C (10.3%);MELD< 10 (52.9%),10–14 (20.6%),> 14 (26.5%)	HCV (90%),MASLD (10%)	LAVI, E, A, e′ (septal/lateral), a′ (septal/lateral), E/A, E/e′	No significant association between LA parameters and severity scores	Not reported
5	Skouloudi et al.,2023,Greece [22]	2018-2022	135-cirrhotic patients	Prospective cross-sectional	CTP: 7.5 ± 2.3; MELD ≥15 (34.8%)	Viral (24.4%),alcohol (27.4%), NAFLD/NASH (7.4%),cryptogenic (8.9%),PSC/PBC (17%),others (14.9%)	LAVI, LA strain (reservoir, conduit, contractile), E/A, E/e′, DT	Higher LAVI in MELD ≥15; reservoir strain <39% linked to poor survival	LA strain and LAVI predict mortality and LT
6	Chigullapalli et al.,2023,India [23]	July 2021-September 2022	50-cirrhotic patients50-controls	Prospectivecase-control	MELD: <9 (18), 10–19 (19), >20 (13)	Alcohol (76%), HBV (10%), HCV (6%), Wilson’s (4%)	LAD, E, A, e′ (septal/lateral), a′ (septal/lateral), E/A, E/e′, DT	Higher MELD score associated with increased LAD and reduced E/A	Not reported
7	Poojary et al.,2022,India [24]	January 2020-January 2021	70- cirrhotic patients 62-controls	Prospective case-control	CTP: A (16), B (54);MELD> 15.5(35)	Not available	LAV, E, A, e′, a′, E/A, E/e′	Increased LAV and E velocity in cirrhotic patients	Not reported
8	Ozersari et al., 2021,Turkey [25]	October 2017 - November 2020	50-cirrhotic patients 4-controls	Prospectivecase-control	CTP: median 6; MELD: median 10	Viral (22),cryptogenic (11),alcohol (8),fatty liver (5),autoimmune (4)	LAD, LAV, LAVI, LAEF, E, A, e′, E/A, E/e′	Increased LAV and impaired LA reservoir function	3D-LAVImin >15 mL/m² associated with poor survival
9	Von Köckritz et al.,2021,Germany [26]	2013-2016	80-cirrhotic patients 30-controls	Retrospective cross-sectional	MELD: 17 ± 6.65	Idiopathic (7),alcoholic (25),HCV (10), NASH (8), PSC (7),autoimmune (8),others (15)	LAVI, LA strain & strain rate, E/A, E/e′, DT	Reduced LA strain in ESLD vs controls	LA strain useful for prognosis and pre-LT assessment
10	Dong Y et al.,2019,China [27]	October 2016-August 2018	67- cirrhotic patients30-controls	Prospective cross-sectional	Not available	NAFLD (67)	LAVI, E, A, e′, a′, E/A, E/e′	Reduced e′, increased E/e′ and LAVI in severe NAFLD	Not reported
11	Lee et al.,2018,Korea [28]	April 2013- April 2015	70-cirrhotic patients	Prospective follow-up	CTP: A (25.7%), B (50%), C (24.3%); MELD: 14.1 ± 5.9	HBV (15),HCV (1),alcohol (47),others (7)	LAVI, e′ (septal/lateral), E/e′, E/A, DT, IVRT	LVDD is present in 62.9%; associated with mortality	LVDD and CTP predict survival
12	Cesari et al.,2018,Italy [29]	2009-2012	115-cirrhotic patients	Prospective cohort	Not available	Alcohol (46%),viral (44%), others (10%)	LAD, E/A, E/e′	Subclinical cardiac dysfunction affects survival	LAD and E/e′ predict mortality
13	Voiosu et al., 2018, Denmark [30]	September 2014-November 2016	58-cirrhotic patients	Prospective observational	CTP A (15%), B/C (85%)	Alcoholic (37),viral (3),autoimmune hepatitis (2),others (6)	LAV, LAVI, E, A, e′, a′, E/A, DT, IVRT	Increased LAV associated with bile acids and hyperdynamic circulation	Not reported
14	Wiese et al.,2018,Denmark [31]	September 2014-February 2017	63- cirrhotic patients 14-controls	Prospective cohort	CTP: A (9), B (46), C (8); MELD median 10	Alcoholic (50),non-alcoholic (11),combined (2)	LAVI, E, A, e′, a′, E/A, E/e′	LA enlargement associated with clinical deterioration	LA enlargement predicts worsening
15	Merli et al.,2017,Italy [32]	Not available	90-cirrhotic patients	Prospective cohort	CTP A/B (74), C (16); MELD: 11.9 ± 4.7	Alcohol (28),viral (49),others (13)	LAVI, E/A, E/e′, DT	Increased LAVI is associated with mortality	Increased LAVI and reduced LVMI independently predicted lower 2-year survival
16	Dinic-Radovic et al.,2017,Serbia [33]	January 2012 -December 2014	40-cirrhotic patients40-controls	Prospectivecase-control	CTP C (70%), B (30%)	Alcohol (80%)	LAD, E/A, DT, IVRT	BNP correlated with LAD	LAD predicts CCM
17	Kumar A et al.,2017,India [34]	Not available	70-cirrhotic patients 20-controls	Prospective case-control	CTP A (17), B (22), C (31); MELD median 14	Alcohol (20), HBV (19,HCV (18),cryptogenic (13)	E/e′, MPI, LV dimensions	DD is present in 64.2% of patients	Not reported
18	Hammami et al.,2017,Tunisia [35]	January 2013-December 2015	80-cirrhotic patients 80-Controls	Prospectivecross-sectional	CTP A (24), B (36), C (20)	Viral (42),cryptogenic (21),others (17)	LA area, E, A, e′, a′, E/A, E/e′, IVRT	LA size correlated with disease severity	Not reported
19	Nath et al.,2016,Bhopal [36]	December 2014-December 2015	60-cirrhotic patients	Prospective observational	CTP B (36.7%), C (63.7%); MELD 20.47 ± 8.92	Alcohol (33.33%),viral (25%),both (13.33%), cryptogenic (28.3%)	LAD, E/A	Higher MELD associated with increased LAD	Not reported
20	Li et al.,2014,China [37]	June 2012-June 2014	89-cirrhotic patients30-controls	Prospectivecase-control	MELD ≤9 (32), 10–19 (34), ≥20 (23)	HBV (56),HCV (10),HBV/HCV (4),alcohol (9),others (10)	LAD, E/A, DT	Higher MELD associated with increased LAD and DT prolongation	Not reported

*Abbreviations*: *3D LAVImin* Three-Dimensional  Left Atrial Volume Index minimum, *A* Late Diastolic Mitral Inflow Velocity, *a*′ Late Diastolic Mitral Annular Velocity, *BNP* B-type Natriuretic Peptide, *CCM* Cirrhotic Cardiomyopathy, *CTP* Child–Turcotte–Pugh Score, *DD* Diastolic Dysfunction, *DT* Deceleration Time, *E* Early Diastolic Mitral Inflow Velocity, *E/A* Ratio of Early to Late Mitral Inflow Velocity, *E/e*′ Ratio of Early Mitral Inflow Velocity to Mitral Annular Velocity, *e*′ Early Diastolic Mitral Annular Velocity, *ESLD* End-Stage Liver Disease, *HBV* Hepatitis B Virus, *HCV* Hepatitis C Virus, *IVRT* Isovolumetric Relaxation Time, *LA* Left Atrium, *LAD* Left Atrial Diameter, *LAEF* Left Atrial Ejection Fraction, *LAV* Left Atrial Volume, *LAVI* Left Atrial Volume Index, *LT* Liver Transplantation, *LVDD* Left Ventricular Diastolic Dysfunction, *LVMI* Left Ventricular Mass Index, *MASLD* Metabolic Dysfunction–Associated Steatotic Liver Disease, *MELD* Model for End-Stage Liver Disease, *MPI* Myocardial Performance Index, *NAFLD* Non-Alcoholic Fatty Liver Disease, *NASH* Non-Alcoholic Steatohepatitis, *n* Number of Participants, *PBC* Primary Biliary Cholangitis, *PSC* Primary Sclerosing Cholangitis

In terms of echocardiographic methodology:

1. Conventional two-dimensional (2D) echocardiography with Doppler assessment was used in all studies.
2. STE for atrial strain analysis was specifically utilized in a subset of studies [22, 26].
3. 3DE was applied in selected studies for the LA volumetric and functional assessment [25].

Diagnostic criteria for CCM and DD varied across studies, with earlier studies relying on the 2005 WCG criteria and more recent investigations incorporating the updated CCC 2019 definitions [10, 38]. This contributed to variability in reported prevalence and associations.

### Aetiology of liver cirrhosis

Aetiology varied from viral infection, alcoholism, nonalcoholic fatty liver disease / nonalcoholic steatohepatitis, metabolically-dysfunction-associated steatotic liver disease (MASLD), cryptogenic/primary biliary cholangitis, and autoimmune. Alcoholism-induced liver cirrhosis is the most observed aetiology in most of the studies. Both CTP and MELD scores assessed in the literature were included in the summary.

### Key findings related to the LA function and cirrhosis

#### LA structural parameters

LAD, LAV, and LAVI were the principal structural parameters assessed. Across studies, LA enlargement was a frequent finding in cirrhotic patients. Increased LAD was consistently associated with worsening liver disease severity, particularly with higher MELD and CTP scores [23, 35–37]. LAD also demonstrated correlation with elevated brain natriuretic peptide levels, suggesting a link between atrial remodeling and neurohormonal activation [33]. LAVI was significantly higher in patients with advanced liver disease in several studies [30]. Skouloudi et al. reported higher LAVI in patients with MELD scores ≥ 15 [22]. Merli et al. identified increased LAVI as an independent predictor of 2-year mortality [32]. Wiese et al. demonstrated that progressive cirrhosis was associated with increasing LAVI over time [31]. In contrast, Mansoor et al. did not observe a significant association between LA volumetric parameters and liver disease severity, highlighting heterogeneity [21]. 3DE assessment provided additional prognostic value. Ozersari et al. reported that a three-dimensional left atrial volume index minimum (3D-LAVImin) > 15 mL/m² was associated with poorer survival, indicating incremental value over conventional measurements [25]. Overall, most studies demonstrated a positive association between LA enlargement and disease severity; however, its direct relationship with elevated filling pressures appeared inconsistent, suggesting contributions from hyperdynamic circulation.

#### LA functional parameters

LA strain parameters, including reservoir, conduit, and contractile strain, were evaluated using STE in selected studies. Reduced LA reservoir and conduit strain were consistently observed in patients with advanced cirrhosis. Von Köckritz et al. demonstrated significantly impaired LA reservoir and conduit strain and strain rate in end-stage liver disease compared with controls [26]. Skouloudi et al. identified LA reservoir strain < 39% as associated with worse survival outcomes [22]. These findings suggest that the LA strain may be more sensitive than volumetric indices alone for detecting subclinical dysfunction. Importantly, strain abnormalities were observed even when conventional systolic parameters such as left ventricular ejection fraction (LVEF) were preserved.

#### Diastolic function parameters

DD was evaluated across studies using early diastolic mitral inflow velocity (E), late diastolic mitral inflow velocity (A), ratio of early to late mitral inflow velocity (E/A ), early diastolic mitral annular velocity (e′), ratio of early mitral inflow velocity to mitral annular velocity ( E/e′ ), deceleration time (DT), and isovolumetric relaxation time (IVRT). Commonly reported abnormalities included:

- Reduced E/A ratio [23, 37].
- E/A ≤ 0.8 is independently associated with increased mortality [20].
- Elevated E/e′ ratio, with E/e′ ≥10 associated with reduced survival [28].
- Decreased septal and lateral e′ velocities [27].

Several studies identified DD as an independent predictor of adverse outcomes, including mortality and post-LT complications [18–20, 28, 29, 34].

However, discrepancies were observed. Some studies did not demonstrate a clear prognostic impact of systolic or DD in stable cirrhotic outpatients, suggesting that patient selection and disease stage significantly influence observed associations [21, 24].

### Prognostic associations

Altered LA structure and function consistently emerged as markers of adverse outcomes.

Independent prognostic predictors included:

- Increased LAVI [32].
- LA reservoir strain < 39% [22].
- 3D-LAVImin > 15 mL/m² [25].
- E/A ≤ 0.8 [20].
- Elevated E/e′ ≥10 [28].

Several studies demonstrated associations between LA parameters and mortality, LT, or clinical deterioration [22, 25, 31–33]. Nonetheless, heterogeneity in methodology, cut-off values, and diagnostic definitions limits direct comparison across studies.

### Consistency and heterogeneity across studies

While most studies demonstrated an association between LA enlargement or impaired atrial strain and worsening liver disease severity, the magnitude of this association varied across studies:

- The strength of correlation with MELD and CTP scores [23, 37].
- The independence of prognostic associations after adjustment [22, 28, 29, 32].
- The prevalence of DD depends on the criteria applied [18].

Differences in diagnostic definitions (2005 WCG vs. 2019 CCC), imaging techniques (2D vs. STE vs. 3DE), and study populations likely contributed to variability in findings. Despite this heterogeneity, the overall body of evidence supports a consistent pattern of structural remodeling and functional impairment of the LA in cirrhosis, with emerging evidence favoring strain-based assessment for improved sensitivity and prognostic stratification [22, 26].

## Discussion

This is the first scoping review appraising the broad range of LA function using echocardiography to detect subtle CCM. Across the 20 included studies in this review, LA structural enlargement, impaired atrial deformation, and diastolic abnormalities were recurrent findings in patients with cirrhosis. CCM is characterized by impaired myocardial contractility and altered diastolic relaxation; however, our synthesis specifically demonstrates that LA parameters were frequently associated with liver disease severity and clinical outcomes within the included cohorts.

A notable observation across the included studies was the variability in diagnostic criteria applied for CCM and diastolic dysfunction. Studies using revised criteria reported lower prevalence compared with earlier definitions [18]. This variability limits direct comparison across cohorts and partly explains the wide range of reported prevalence.

### LA enlargement

Studies included in this review consistently reported LA enlargement in cirrhotic patients. Hammami et al., Li et al., Chigullapalli et al., and Nath et al. demonstrated positive correlations between LA size (LAD or LAVI) and liver disease severity, as measured by MELD or Child–Pugh scores [23, 35–37]. Skouloudi et al. reported higher LAVI in patients with MELD scores ≥ 15, and Lupu et al. observed greater LAVI in Child–Pugh C patients [19, 22]. In addition to disease severity, LA enlargement was linked to outcomes. Merli et al. identified increased LAVI as an independent predictor of 2-year mortality [32]. Ozersari et al. demonstrated that 3D-LAVImin > 15 mL/m² was associated with poorer survival [25]. Cesari et al. and Lee et al. reported associations between diastolic abnormalities and mortality [28, 29]. However, associations were not uniform across all cohorts. Mansoor et al. did not observe significant correlations between LA parameters and severity indices, highlighting inter-study heterogeneity [21].

### LA strain

Beyond volumetric assessment, STE was utilized in selected studies. Von Köckritz et al. demonstrated significantly reduced LA reservoir and conduit strain in end-stage liver disease [26]. Skouloudi et al. identified LA reservoir strain < 39% as associated with worse survival outcomes [22]. These findings within the included studies suggest that strain-derived indices may detect functional impairment even when conventional systolic parameters remain preserved. However, strain analysis was not uniformly applied across all studies, limiting comparability.

### DD as a primary feature

DD was frequently reported, though prevalence varied across criteria. Voet et al. reported a lower prevalence when revised criteria were applied [18]. Li et al. and Chigullapalli et al. reported a reduced E/A ratio with increasing MELD scores [23, 37]. Wang et al. identified E/A ≤ 0.8 as independently associated with mortality [20]. Lee et al. reported that E/e′ ≥10 was associated with lower survival [28]. Differences in parameter definitions, acquisition techniques, and applied cut-off values contributed to variability in reported prevalence and prognostic strength across studies.

### Prognostic significance

Across the included studies, altered LA structure and function were repeatedly associated with adverse outcomes. Increased LAVI, reduced LA reservoir strain < 39%, elevated 3D-LAVImin, and abnormal diastolic indices emerged as markers of poor prognosis [20, 22, 25, 28, 32]. Nevertheless, all included studies were observational, and none were randomized. Sample sizes varied considerably, aetiologies of cirrhosis differed across cohorts, and follow-up duration was inconsistent. These methodological differences limit the ability to draw uniform conclusions regarding the magnitude of prognostic impact.

Furthermore, interpreting echocardiographic parameters in cirrhosis is inherently complex. Doppler-derived indices (E/A, E/e′), tissue Doppler velocities, volumetric indices, and strain measurements each have intrinsic methodological limitations. In cirrhosis, these limitations are further amplified by hyperdynamic circulation, altered preload and afterload conditions, and fluctuations in intravascular volume status, which may independently influence measurements beyond intrinsic myocardial dysfunction.

The 2025 American Society of Echocardiography guidelines emphasize an integrative, multiparametric approach to the assessment of diastolic and atrial function, particularly in complex clinical settings [39]. In patients with cirrhosis, contextual interpretation of structural and functional indices, rather than reliance on a single parameter, is essential to avoid misclassification. Overall, while LA enlargement and impaired atrial mechanics were frequently observed across the included studies and demonstrated potential prognostic relevance, substantial heterogeneity in methodology and diagnostic frameworks underscores the need for standardized, prospective investigations applying contemporary guideline-based criteria.

### Clinical implications

Given the high prevalence and prognostic significance of altered LA function and DD reported across the included studies, routine echocardiographic evaluation should be considered in patients with cirrhosis, irrespective of apparent liver disease severity. This is particularly important prior to interventional procedures such as transjugular intrahepatic portosystemic shunt insertion or LT, where underlying cardiac dysfunction may increase peri-procedural risk. Clinicians should consider incorporating advanced echocardiographic techniques, including STE for LA strain assessment and 3DE for volumetric analysis, as several included studies demonstrated their incremental prognostic value. Parameters such as increased LAVI, reduced LA reservoir strain, and elevated 3D-LAVImin were associated with adverse outcomes, including mortality and need for transplantation. However, interpretation of these parameters must account for the heterogeneity of diagnostic criteria and for the influence of hyperdynamic circulation and altered loading conditions, which are commonly observed in cirrhosis. A multiparametric and guideline-based approach is recommended to improve diagnostic accuracy and risk stratification.

Beyond established cirrhosis, emerging evidence suggests that STE may detect subclinical myocardial dysfunction at earlier stages of chronic liver disease, including MASLD. Although most available data focus on ventricular strain, these observations support consideration of earlier cardiovascular risk stratification in metabolically high-risk populations. Notably, a recent meta-analysis demonstrated the incremental diagnostic and prognostic utility of STE-derived myocardial strain parameters in MASLD patients, underscoring its ability to identify early cardiac impairment [40]. Early identification of diastolic dysfunction, particularly features consistent with heart failure with preserved ejection fraction (HFpEF), may have implications for broader cardiovascular risk assessment in patients with cirrhosis. Although SGLT2 inhibitors have demonstrated benefit in HFpEF populations, direct evidence supporting their use in CCM remains limited. The potential impact of existing medications, including beta-blockers and diuretics, on cardiac function and echocardiographic parameters in this population also warrants careful evaluation. Overall, integrating structural and functional LA assessment into routine clinical evaluation may improve the identification of high-risk patients and guide individualized management strategies.

### Strengths and limitations of the current review

This is the first scoping review that evaluates LA function in the context of CCM. We have strictly followed the JBI guidelines and the PRISMA-ScR standards. During the pilot phase of our search strategy, we ensured it was effective in gathering key papers deemed highly relevant. A total of 364 articles were screened by at least two independent reviewers, who then conducted a thorough full-text review of the potential articles for inclusion. By synthesizing structural, functional, and strain-based parameters across diverse study designs, this review provides a consolidated overview of the available evidence and identifies key methodological variability and research gaps.

Our review has certain limitations. We primarily focused on identifying literature related to LA function in patients with chronic liver disease. Consequently, we did not include other parameters, such as ventricular function assessment. We specifically concentrated on LA function rather than diastolic function assessment, as including the latter would have complicated our search strategy and broadened the scope beyond our predefined objectives. As this study was a scoping review, formal assessment of study quality, risk of bias, or publication bias was not conducted. The objective was to map and synthesize available literature rather than to conduct quantitative pooling or meta-analysis. Additionally, the included studies were highly heterogeneous in design, patient populations, aetiology of cirrhosis, echocardiographic methodology, and outcome assessment. All studies were observational, and none were randomized, limiting causal inference. Variability in diagnostic criteria for CCM and DD further complicates direct comparison across cohorts. Furthermore, echocardiographic indices used to assess diastolic and atrial function have inherent methodological limitations, which may be influenced by hyperdynamic circulation and altered loading conditions commonly observed in cirrhosis. These physiological alterations may affect Doppler- and strain-derived measurements independently of intrinsic myocardial dysfunction. Although our strategy identified some articles published in multiple languages, we excluded non-English articles, which may have limited comprehensiveness.

## Conclusion

This scoping review highlights the role of echocardiographic assessment of LA function in detecting CCM. Structural changes, such as atrial enlargement and increased volume, along with reduced strain, are frequently observed in cirrhotic patients and correlate with disease severity and can be considered a marker of subtle LV DD in early-onset CCM. These parameters also serve as prognostic markers, predicting adverse outcomes including mortality and post-transplant complications. Advanced echocardiographic modalities, particularly STE and 3DE, demonstrate superior sensitivity in identifying subclinical dysfunction compared with conventional methods. Their integration into routine evaluation could improve risk stratification, especially in patients awaiting LT or undergoing invasive procedures. However, heterogeneity in diagnostic criteria and methodologies across studies underscores the need for standardized definitions and prospective validation. Overall, assessing LA function offers valuable clinical insights, and future research should focus on refining diagnostic frameworks to enable earlier detection and optimized management of CCM.

## Supplementary Information

Supplementary Material 1.

Supplementary Material 2.

## Data Availability

No datasets were generated or analysed during the current study.

## Abbreviations

2D: Two-dimensional
3DE: Three-dimensional echocardiography
3D-LAVImin: Three-dimensional left atrial volume index minimum
A: Late diastolic mitral inflow velocity
CCM: Cirrhotic cardiomyopathy
CCC: Cirrhotic cardiomyopathy consortium
CTP: Child–Turcotte–Pugh score
DD: Diastolic dysfunction
DT: Deceleration time
E: Early diastolic mitral inflow velocity
E/A: Ratio of early to late mitral inflow velocity
E/e′: Ratio of early mitral inflow velocity to mitral annular velocity
e′: Early diastolic mitral annular velocity
IVRT: Isovolumetric relaxation time
JBI: Joanna Briggs institute
LA: Left atrium
LAD: Left atrial diameter
LAV: Left atrial volume
LAVI: Left atrial volume index
LT: Liver transplantation
LV: Left Ventricle
LVEF: Left ventricular ejection fraction
MASLD: Metabolic dysfunction–associated steatotic liver disease
MELD: Model for end-stage liver disease
PCC: Population, concept, context
PRISMA-ScR: Preferred reporting items for systematic reviews and meta-analyses extension for scoping reviews
STE: Speckle tracking echocardiography
WCG: World congress of gastroenterology
