# Supplementary material for: Assessing the left atrial function via echocardiography to detect cirrhotic cardiomyopathy in patients with liver cirrhosis - a scoping review
Source: Cardiovasc Ultrasound. 2026 Oct 1;24:23. doi: 10.1186/s12947-026-00383-5 (PMC13628834; doi:10.1186/s12947-026-00383-5)
Supplement: Supplementary file 2 [file 12947_2026_383_MOESM1_ESM.docx]

## Title

Assessing the left atrial function via echocardiography to detect cirrhotic cardiomyopathy in patients with liver cirrhosis: A scoping review

## Background & Introduction

Cirrhotic cardiomyopathy (CCM) is a chronic cardiac dysfunction in cirrhotic patients, marked by stress-induced systolic impairment, altered diastolic relaxation, and electrophysiological changes. Initially defined at the 2005 World Congress of Gastroenterology, CCM criteria were updated in 2019 by the Cirrhotic Cardiomyopathy Consortium to incorporate advanced echocardiographic methods such as tissue Doppler imaging and myocardial strain.Early detection of cardiac changes is critical for managing CCM and improving outcomes, particularly in liver transplant candidates. Advanced modalities like speckle-tracking and 3D echocardiography provide a more comprehensive evaluation of both systolic and diastolic function, offering deeper insight into cardiac involvement in cirrhosis. A scoping review is therefore appropriate to summarize available evidence, highlight research gaps, and guide future studies and clinical practice in the management of CCM.

## Review objectives

This scoping review aims to systematically map the literature on echocardiographic assessment of LA function in liver cirrhosis patients. It outlines the parameters used, their association with cirrhosis severity, and their prognostic value.

Using the PCC framework

Population:

The scoping review focused on adult patients with liver cirrhosis

Concept:

Assessment of left atrial function via echocardiography

Context: detecting cirrhotic cardiomyopathy

Review question: How does echocardiographic assessment of left atrial function contribute to the early detection and characterization of cirrhotic cardiomyopathy in patients with liver cirrhosis?

## Inclusion criteria

Population:

The scoping review focused on adult patients with liver cirrhosis

Concept:

Assessment of left atrial function via echocardiography

Context:

Detecting cirrhotic cardiomyopathy in a health setting

type of sources:

mixed methods

## Methods

### -Search strategy

Initial limited search-

Liver cirrhosis, Chronic liver disease, left atrial function, strain echocardiography, Cirrhotic cardiomyopathy, Left ventricular dysfunction

List databases to be searched-PubMed, Scopus, Web of Science

Grey literature: Nil

Use of librarian/information specialist: PubMed, Scopus, and Web of Science

A sample strategy for at least one database will be represented in the appendix

### -Study selection

Title/abstract and full text screening will be done by two reviewers at two levels of screening. If any disagreement arises in the selection process, it will be resolved by consensus agreement. Rayyan software will be used for screening and full-text screening of the article.

### -Data extraction (Charting the data)

The data extraction chart will include specific details about the first author, year, Country, Sample numbers, methodology, CTP score, MELD score, Etiology of Cirrhosis, left atrial function parameter assessed, key findings related to LA function and CCM, and Cardiac prognostic outcome. The form pilot will be tested. If any discrepancies arise during the data extraction, they will be resolved by discussion or with an additional reviewer.

### -Data analysis and presentation

Descriptive Summary of the data will be represented by Tables.

## Ethics and dissemination

Ethical approval is not applicable as human participants or identifiable data are not used in the study. Plans for dissemination will be a journal publication.

## References

As per the target journal reference will be put

## Appendices

Search strategy:

("Liver Cirrhosis" OR "Liver Diseases" OR "Chronic liver disease" OR "Decompensated Chronic liver disease") AND ("atrial function, left" OR Echocardiography OR "echocardiography, three dimensional" OR "atrial function, left" OR "Left atrial strain" OR "Left atrial reservoir strain" OR "Strain echocardiography") AND ("Cirrhotic cardiomyopathy" OR CCM OR "ventricular dysfunction, left")

Data charting form:

| **SL. No** | **First Author and Year** | **Study period** | **N** | **Methodology** | **Child Pugh Score**  **MELD score**  **(Mean/median/%)** | **Etiology of cirrhosis** | **LA function parameters assessed** | **Key Findings Related to LA Function and CCM** | **Cardiac prognostic outcome** |
| --- | --- | --- | --- | --- | --- | --- | --- | --- | --- |
| **1** | Voet [et.al](http://et.al/)  2025  Belgium [41] | January 2005 to March 2017 | 233 | Retrospective cohort | Median  Child-Pugh score  A: 36  B: 96  C: 86  Median MELD score: 17 (11) | Alcoholic liver disease – 116  Autoimmune and biliary disease - 37  HBV – 23  HCV – 33  MASLD - 22 | E, A, E' (IVS & LW), A' (IVS &LW), E/A, E/E' | DD prevalence is lower with the revised criteria | LA function and reduced e’ lateral velocity associated with post-LT mortality |
| **2** | Lupu, Nedelcu, and Tint  2024  Romania  [35] | November 2021 to March 2024 | 102 | Prospective observational study | Child A&B n = 65 Child C n = 37 | Alcohol (77)  viral etiology (18)  Other (7) | LAVi  E/e' | Significantly greater burden of DD in severe cirrhosis vs milder | DD correlated with the severity of portal hypertension, reflected by the portal vein and spleen diameter sizes. |
| **3** | Wang et al,  2024.  China.[36] | 22 June 2020 to 30 June 2022 | 104 | Retrospective cohort study | Child Pugh score (7.63 ± 2.18)  Child Pugh A/B- 37 (35.6%)  Child Pugh C- 67 (64.4%)  MELD Score:  9.94 (6.43–35.74)  11.94 ± 5.46 | HBV infection - 30 (28.8%)  HCV infection - 6 (5.8%)  Alcohol abuse - 47 (45.2%) | LA dimension  E, A, E/A | E/A ≤ 0.8 was independently associated with a higher risk of death in cirrhotic patients. | E/A ≤0.8 is significantly associated with higher cumulative death incidence. |
| **4** | Mansoor [et.al](http://et.al/)  2024  Pakistan [42] | August 2021-June 2023 | 68 | Prospective cross-sectional | Class A -32.4% Class B -57.4% Class C -10.3%  MELD < 10 -52.9% 10–14 -20.6% > 14 -26.5% | Hepatitis C 90% MASLD 10% | LA vol index  E, A, E' (IVS & LW), A' (IVS &LW), E/A, E/E' | No significant relationship between LA function parameters and liver disease severity scores | - |
| **5** | Marina Skouloudi et.al,  2023  Greece  [29] | 2018-2022 | 135 | Prospective Cross-sectional | CTP 7.5 ± 2.3(mean ± SD)  MELD <15 (65.2%) MELD score ≥15 (34.8%) | Viral hepatitis (24.4%)  Alcohol (27.4%) NAFLD/NASH (7.4%)  Cryptogenic (8.9%)  PSC/PBC (17%),  Other (14.9%) | LAD, LAV, LAVI, LA reservoir strain, LA conduit strain, LA contractile strain,  E' septal/E’, E/A, DT | Higher LAVI with MELD ≥15.  Reservoir strain <39% linked to worse survival. | LA strain parameters and LAVI associated with death and liver transplantation |
| **6** | Chigulla Palli et al  2023  India  [25], | July 2021 to September 2022 | 50 – case  50 - control | Prospective Case–Control | MELD  < 9 = 18  10-19= 19  >20 = 13 | alcoholic cirrhosis (76%), hepatitis B virus infection (10%), hepatitis C virus  infection (6%), and Wilson’s disease in 4% | LA diameter  E, A, E' (IVS & LW), A' (IVS &LW), E/A, E/E', DT | Increasing MELD score correlated with significant increases in LA diameter and decreased E/A ratio. | - |
| **7** | Poojary [et.al](http://et.al/),  2022  India  [30] | January 2020 to January 2021 | 70 – case  62 - controls | Prospective case-control | Child-Pugh A - 16, Child-Pugh B – 54  MELD <15.5: 35, MELD> 15.5: 35 | NA | Left atrial volume  E, A, E' (IVS & LW), A' (IVS &LW), E/A, E/E' | LA volume and Mitral E velocity were significantly higher in the cirrhosis group than in the control group. | - |
| **8** | Ozersari et.al, 2021  Turkey  [10], | October 2017 - November 2020 | 50 case 43 control | Prospective Case Control | CTP median 6.0 (5–13)  MELD 10.0 (6–29) | Viral (22),  cryptogenic (11),  alcoholic (8),  fatty liver (5),  autoimmune (4) | LAD, LAV, LAVI; 3D parameters of LAV and LAEF; E velocity, A velocity, Em velocity, E/A, E/Em | Elevated LA volumes and impaired LA reservoir function in cirrhosis. | 3D-LAVImin >15 ml/m2 linked to poorer survival |
| **9** | Von Köckritz et al  2021  Germany  [34] | 2013-2016 | 80 Cirrhosis, 30 controls | Retrospective cross-sectional | MELD 17 ± 6.65 | Idiopathic (7) Alcoholic (25)  HCV (10)  NASH (8)  PSC (7)  Autoimmune (8)  Others (15) | LAVi  reservoir, conduit & contractile strain  reservoir, conduit & contractile strain rate  E/A, E/e', DT | significantly reduced LA reservoir and conduit strain and strain rate in all phases in end-stage liver disease patients versus controls. | LA strain parameters may guide prognosis and pre-liver transplant cardiac assessment. |
| **10** | Dong Y [et.al](http://et.al/)  2019  China  [37] | October 2016  and August 2018 | 67- case  30 controls | Prospective Cross-sectional | NA | NAFLD (67) | LA Max vol index  E, A, E' (IVS & LW), A' (IVS &LW), E/A, E/E' | Decreased septal/lateral e' velocities, increased E/e', and LAVImax in the moderate-to-severe NAFLD group. | - |
| **11** | Lee et.al,  2018  Korea  [38] | April 2013 to April 2015 | 70 cases | Prospective follow-up study | Child-Pugh Class A (25.7%)  B (50.0%)  C (24.3%)  MELD 14.1±5.9 | HBV (15)  HCV (1)  Alcohol (47)  others (7) | LAVI  E' septal & Lateral, E/E’, E/A, DT, IVRT | 62.9% had LVDD, and there was a higher mortality with LVDD.  Child-Pugh and LVDD were independent predictors of survival.  E/e’ ≥10 linked to lower survival | LVDD and Child-Pugh score predict survival |
| **12** | Cesari et al,  2018  Italy  [39] | 2009-2012  (6-year follow-up) | 115 | Prospective Cohort Study | NA | Alcoholic cirrhosis (46%),  Post-viral cirrhosis (44%), Other causes (10%) | LAD  E/A. E/e', | Subclinical cardiac morphologic and functional changes can influence survival in cirrhosis. | Left atrial dimension, increased E/e’ ratio were independent predictors of death in cirrhotic patients during follow-up. |
| **13** | Voiosu et.al  [2018](http://et.al/)  Denmark  [32] | September 2014 to November 2016 | 58 | Prospective observational | Class A-15%  Class B/C-(85%) | Alcoholic -37  Viral – 3  Autoimmune hepatitis – 2  cryptogenic or other - 6 | LA Vol/ vol index  E, A, E' (IVS & LW), A' (IVS &LW), E/A, E', DT, IVRT | Total serum bile acid levels are associated with larger left atrial volume and hyperdynamic circulation markers. | Serum bile acid levels correlate with left atrial enlargement and hyperdynamic circulation in cirrhosis, independent of etiology and disease severity. |
| **14** | Wiese et.al  2018  Denmark [43] | September 2014 to February 2017 | 77 (63 - case, 14 - controls) | Prospective cohort | Child–Pugh A = 9, B = 46, C = 8  MELD -10 (8, 13) | Alcoholic -50  Non-Alcoholic -11  Combined - 2 | LAVI  E, A, E' (IVS & LW), A' (IVS &LW), E/A, E/E', | Patients progressing to acute decompensation, LT, or death exhibited more left atrial enlargement and myocardial structural changes. | Left atrial enlargement was more pronounced in patients who worsened clinically. |
| **15** | Merli et al  2017  Italy  [33] | NA | 90 | Prospective Cohort Study | Child A-B (74)  Child C (16)  MELD 11.9 ± 4.7 | Alcohol (28)  Viral (49)  other (13) | LAVi  E/A, E/e’, DT | An increased left atrial volume and a lack of compensatory left ventricular hypertrophy is associated with higher mortality at 2 years. | Increased LAVi and decreased LVMi independently predicted lower 2-year survival. |
| **16** | Dinic-Radovic et al,  2017  Serbia  [28] | January 2012 and December 2014 | Cirrhosis 40  Control 40 | Prospective case control | Child C (70%)  Child B (30%) | Alcoholic liver cirrhosis (80%) | LAD  E/A, DT, IVRT | Significant elevation of BNP correlated with left atrial diameter. | LA diameter is a significant predictor of CCM. |
| **17** | Kumar A et al  2017  India  [40] | NA | case 70  control 20 | Prospective case control | Child A=17 Child B=22 Child C=31  Median MELD score 14 (3 - 27) | Alcohol (20) Hepatitis B (19) Hepatitis C (18) Cryptogenic (13) | E: E's ratio, E: E’L, myocardial performance index, LV diastolic and systolic dimensions | 64.2% of cirrhotics had DD by E/E’s cut off <8; the median E deceleration time differed significantly between cirrhotics and controls | Cirrhotic patients demonstrated significant DD with normal systolic function. |
| **18** | Hammami [et.al](http://et.al/)  2017  Tunisia [26] | January 2013 and December 2015 | 80 - case  80- Controls | Prospective, cross-sectional | Child A – 24  Child B – 36  Child C - 20 | Viral (hepatitis B.C): 42  Cryptogenic: 21  Other causes: 17 | LA Area  E, A, E' (IVS & LW), A' (IVS &LW), E/A, E/E', IVRT | LA size correlated positively with Child and MELD scores | - |
| **19** | Nath Dubey and Panjwani et al,  2016  Bhopal  [44] | December 2014  to December 2015 | 60 | Prospective observational study | Child B (36.7%)  Child C (63.7%)  MELD 20.47±8.92 | Alcoholic (33.33%)  Viral (25%)  Both (13.33%)  Cryptogenic (28.3%) | LAD  E/A | Patients with more severe liver disease (MELD >16) were found to have higher left atrial diameter when compared with patients who had MELD scores <16. | **-** |
| **20** | Li X et al,  2014  China.[27] | June 2012 and June 2014 | Case-89  Control-30 | Prospective Case Control | MELD ≤ 9 (n = 32) MELD 10-19 (n = 34) MELD ≥ 20 (n = 23) | HBV-56  HCV-10  HBV/HCV-4  alcoholic liver disease -9 cholestatic liver disease-8 cryptogenic cirrhosis-2 | Left atrial diameter (LAD), VE/VA ratio  DT | Higher the MELD score higher the LAD, DT Prolongation and LOWER E/A ratio | - |
